# Proteomics and antivenom immunoprofiling of Russell’s viper (*Daboia siamensis*) venoms from Thailand and Indonesia

**DOI:** 10.1590/1678-9199-JVATITD-2019-0048

**Published:** 2020-01-31

**Authors:** Thava Malar Changra Lingam, Kae Yi Tan, Choo Hock Tan

**Affiliations:** 1 Department of Molecular Medicine, University of Malaya, Kuala Lumpur, Malaysia.; 2 Department of Pharmacology, University of Malaya, Kuala Lumpur, Malaysia.

**Keywords:** Venomics, Antivenomics, Eastern Russell's viper, Neutralization

## Abstract

The Eastern Russell’s viper, *Daboia siamensis*, is a WHO Category 1 medically important venomous snake. It has a wide but disjunct distribution in Southeast Asia. The specific antivenom, *D. siamensis* Monovalent Antivenom (DsMAV-Thailand) is produced in Thailand but not available in Indonesia, where a heterologous trivalent antivenom, Serum Anti Bisa Ular (SABU), is used instead. This study aimed to investigate the geographical venom variation of *D. siamensis* from Thailand (Ds-Thailand) and Indonesia (Ds-Indonesia), and the immunorecognition of the venom proteins by antivenoms.

**Methods:**

The venom proteins were decomplexed with reverse-phase high-performance liquid chromatography and sodium dodecyl sulfate-polyacrylamide gel electrophoresis, followed by in-solution tryptic digestion, nano-liquid chromatography-tandem mass spectrometry and protein identification. The efficacies of DsMAV-Thailand and SABU in binding the various venom fractions were assessed using an enzyme-linked immunosorbent assay optimized for immunorecognition profiling.

**Results:**

The two most abundant protein families in Ds-Thailand venom are phospholipase A_2_ (PLA_2_) and Kunitz-type serine protease inhibitor (KSPI). Those abundant in Ds-Indonesia venom are PLA_2_ and serine protease. KSPI and vascular endothelial growth factor were detected in Ds-Thailand venom, whereas L-amino acid oxidase and disintegrin were present in Ds-Indonesia venom. Common proteins shared between the two included snaclecs, serine proteases, metalloproteinases, phosphodiesterases, 5’nucleotidases and nerve growth factors at varying abundances. DsMAV-Thailand exhibited strong immunorecognition of the major protein fractions in both venoms, but low immunoreactivity toward the low molecular weight proteins e.g. KSPI and disintegrins. On the other hand, SABU was virtually ineffective in binding all fractionated venom proteins.

**Conclusion:**

*D. siamensis* venoms from Thailand and Indonesia varied geographically in the protein subtypes and abundances. The venoms, nevertheless, shared conserved antigenicity that allowed effective immunorecognition by DsMAV-Thailand but not by SABU, consistent with the neutralization efficacy of the antivenoms. A specific, appropriate antivenom is needed in Indonesia to treat Russell’s viper envenomation.

## Background

The Russell’s viper is a complex of true viper (subfamily: Viperinae) in Asia, previously recognized as monotypic *Daboia russelii* or *Vipera russelii* with at least seven subspecies: *D. r. russelii* (India), *D. r. pulchella* (Sri Lanka), *D. r. nordicus* (Northern India), *D. r. siamensis* (Indochina), *D. r. formosensis* (Taiwan), *D. r. limitis* (Indonesia) and *D. r. sublimitis* [1]. Mitochondrial DNA and multivariate morphological analyses revealed that Russell’s viper diverged approximately 7-11 mybp (million years before present) into two distinct east-west clades, separated by a narrow range of mountains from northwest Burma to the north of the Bay of Bengal [2]. 

*Daboia russelii* now refers to the western clades of Russell’s viper that inhabits the Indian subcontinent and neighbouring countries including Pakistan, Sri Lanka and Bangladesh. Meanwhile, the subspecies of the eastern clade of Russell’s viper (*D. r. limitis, sublimitis, siamensis* and *formosensis*) have been collectively elevated to full species status, namely *Daboia siamensis* (Eastern Russell’s viper). The distribution of *D. siamensis* is wide but extremely disjunctive. It is found in Guangdong and Guangxi (southern China), and the insular population found in Taiwan marks the easternmost distribution of this species. In the south, it is distributed in Indochina subcontinent covering Myanmar (Burma), Thailand. Laos and western Cambodia but not in Vietnam, and it is absent throughout Malaysian Peninsula, Borneo and the Philippines. Nonetheless, further to the south across the South China Sea and the Javan Sea, the species emerges in isolated populations in the eastern Java and some islets of Lesser Sunda, on the Indonesian Archipelago.

Snake venoms are evolutionary products suited to the survival of the species in adaptation to different ecological niches. Hence, the venom compositions can be highly variable even within the same species due to factors such as geographical, sexual and ontogenic variations [3, 4]. It has been shown in the western Russell’s viper (*D. russelii*) that the venoms from different geographical populations were variable in composition [5-8], and the variation could be the cause of the discrepancy in toxicity and antivenom efficacy. In the context of geographical variation impacting on venom composition, the extreme disjunctive distribution of Indonesian *D. siamensis* is of particular medical interest as it is isolated by more than 2000 km from the nearest conspecific populations in Thailand. Although the current phylogenetics implies no allopatric specification (the Indonesian *D. r. limitis* is the same as the *D. siamensis*, the Eastern Russell’s viper), the possibility of venom variation between the Indonesian and the Thai Russell’s vipers cannot be excluded without a comprehensive comparison of the venom compositions. 

In Indonesia, *D. siamensis* is commonly found in open, grassy areas, rice fields and agricultural areas when prey items are abundant. *D. siamensis* is an ambusher, and its attraction to agricultural and farming zones greatly increases the risk of human envenoming. Although under-reported, Russell’s viper envenoming is not uncommon in Indonesia where the snake is endemic (Java and islets of Lesser Sunda), and patients typically show hematological complications characterized by consumptive coagulopathy and internal bleeding (Tri Maharani, personal communication). Interestingly, variation in the envenoming toxicity of Russell’s viper from different geographical locales are well established, but the differences do not necessarily relate to phylogeny [2, 9]. Clinical studies showed that the Sri Lankan *D. russelii* envenoming commonly causes hemotoxicity and nephrotoxicity in addition to presynaptic neurotoxicity [10, 11]. On the other hand, pituitary hemorrhage along with severe coagulopathy is commonly associated with envenomation by *D. siamensis* in Myanmar [12, 13], whereas *D. siamensis* envenoming in China and Taiwan is associated with coagulopathy and internal bleeding [14-16]. While the evolutionary mechanism awaits further resolution, the phenomenon has a medical implication in which *D. siamensis* venom composition should be investigated based on the distinctive geographical locale from where the venom originates. 

The recent venomic study of Eastern Russell’s viper from Taiwan and China revealed geographical venom variation that supports the clinical observation of envenoming effects [17, 18]. In Southeast Asia, the proteomics of *D. siamensis* venom from Myanmar was previously reported [19]; however, an in-depth venom proteome that details the protein subtypes and expression level for *D. siamensis* originating from Southeast Asia remains to be elucidated. In particular, the profiling of the Thai and Indonesian *D. siamensis* venom proteomes is crucial to determine the potential compositional variation in the venom of the two geographically distant populations. The knowledge will provide valuable information for the subsequent comparison of venom protein antigenicity and immunological reactivity with antivenoms available in the countries. 

The Thai *D. siamensis* monovalent antivenom (DsMAV-Thailand, a product of Queen Saovabha Memorial Institute, Bangkok) is the major antivenom produced against *D. siamensis* envenoming in Southeast Asia, besides the production of *D. siamensis* monovalent antivenom in Myanmar for domestic use. Unfortunately, in Indonesia, there is no locally produced antivenom against the Indonesian *D. siamensis*. This has led to the use of inappropriate, non-specific Indonesian antivenom, Serum Anti Bisa Ular (SABU, indicated for treatment of envenoming by the Javan spitting cobra, banded krait and Malayan pit viper) [20, 21]. A recent study demonstrated that the procoagulant and lethal effects of *D. siamensis* from Indonesia (Ds-Indonesia) venom can be effectively neutralized by DsMAV-Thailand but, importantly, not SABU [22]. This implies that the venoms of *D. siamensis* from Thailand and Indonesia likely share a similar protein and antigenicity profile, despite the disjunctive geographical distribution between the two populations. Hence, this study set to investigate and compare the protein composition of the Indonesian and the Thailand *D. siamensis* venoms through a decomplexing proteomic approach [23], followed by examining the immunorecognition of the various protein components in the venoms by DsMAV-Thailand and SABU. 

## Methods

### Venoms and Antivenoms

*Daboia siamensis* (*D. siamensis*) venom from Thailand was supplied by the Queen Saovabha Memorial Institute (QSMI), Bangkok (pooled from more than five specimens), and the Indonesian *D. siamensis* venom was sourced from Rizki supply at eastern Java (pooled from five adult specimens). All venoms were lyophilized and stored at −20°C prior to use. Two antivenoms were used in this study. The first one was *Daboia siamensis* monovalent antivenom (DsMAV-Thai, batch no. WR00212; expiry date: November 2017, product of Queen Saovabha Memorial Institute in Bangkok), a lyophilized product containing purified F(ab)’_2_ obtained from the sera of horses hyperimmunized against the *D. siamensis* from Thailand. The second antivenom was Serum Anti Bisa Ular (SABU, batch no. 4701516; expiry date: August 2018, product of BioFarma Pharmaceuticals, Indonesia) derived from the sera of horses hyperimmunized against the venoms from three Indonesian snake species: the Javan spitting cobra (*Naja sputatrix*), the Malayan pit viper (*Calloselasma rhodostoma*) and the banded krait (*Bungarus fasciatus*).

### Chemicals and Materials

All chemicals and reagents used in this study were of analytical grade. Ammonium bicarbonate, dithiothreitol (DTT) and iodoacetamide were purchased from Sigma-Aldrich (USA). Mass spectrometry sequencing grade of trypsin proteases, Thermo Scientific Spectra Multicolor Broad Range Protein Ladder (10-260 kDa) and high-performance liquid chromatography (HPLC) grade solvents used in the studies were purchased from Thermo Scientific™ Pierce™ (USA). Millipore ZipTip® C18 Pipette Tips were purchased from Merck (USA).

### C_18_ Reverse-Phase High-Performance Liquid Chromatography (HPLC) 

Crude venom (3 mg/mL) of *D. siamensis* was reconstituted in ultrapure water and centrifuged at 10,000g for 5 minutes. The supernatants were subjected to LiChrospher® WP 300 C_18_ reverse-phase column (5 µm) using a Shimadzu LC-20AD HPLC system (Japan). The venom components were eluted at 1 mL/min with a linear gradient of 0.1% trifluoroacetic acid (TFA) in water as Solution A and 0.1% TFA in 100% acetonitrile as Solution B (0-5% B for 10 min, 5-15% B for 20 min, 15-45% B for 120 min and 45-70% B for 20 min). Proteins were detected by measurement of absorbance at 215 nm and the peaks were collected manually. The fractions were lyophilized and kept in -20ºC until use.

### Sodium Dodecyl Sulfate-Polyacrylamide Gel Electrophoresis (SDS-PAGE) 

The protein fractions obtained from reverse-phase HPLC were further assayed by SDS-PAGE using 15% polyacrylamide gels under reducing condition as per the LaemmLi method [24] calibrated with Thermo Scientific Spectra Multicolor Broad Range Protein Ladder (10-260 kDa). The freeze-dried protein fractions were reconstituted in ultrapure water and 10 µL of each reconstituted fraction were mixed with a sample buffer sample containing mercaptoethanol in 1:1 volume proportion, and heated in boiling water for 15 min. The electrophoresis was performed under reducing conditions at 90 V for 2.5 hours. Upon the completion of SDS-PAGE, the gels were stained using Coomassie Brilliant Blue R-250 and destained overnight. The gel was then visualized for protein bands using Image Scanner III and analyzed using myImage Analysis software (Thermo Scientific). 

### In-solution Tryptic Digestion and Protein Identification by Tandem Mass Spectrometry (nano-ESI- LCMS/MS)

Proteins in the chromatographic fractions (10 µg) for each sample of *D. siamensis* venom (Ds-Thailand and Ds-Indonesia) were subjected to reduction with DDT, alkylation with iodoacetamide and in-solution digestion with mass-spectrometry grade trypsin proteases as described earlier [25]. The digested peptides with trypsin were desalted with Millipore ZipTip C_18_ Pipette Tips. Briefly, the digested peptide eluates were reconstituted in 7 µL of 0.1% formic acid in water. Peptide separation was performed by 1260 Infinity Nanoflow LC system (Agilent, USA) connected to Accurate-Mass Q-TOF 6550 series with a nano-electrospray ionization source. The eluate was subjected to HPLC Large-Capacity Chip Column Zorbax 300-SB-C18 (160 nL enrichment column, 75 µm × 150 mm analytical column and 5 µm particles - Agilent, USA). Injection volume was adjusted to 1 µL per sample, using a flow rate of 0.4 µL/min, with linear gradient of 5-7% of solvent B (0.1% formic acid in 100% acetonitrile). Drying gas flow was 11 L/min and drying gas temperature was 290°C. Fragmentor voltage and capillary voltage was set at 175 V and 1800 V respectively. 

Mass spectra was acquired using mass hunter acquisition software (Agilent, USA) in a MS/MS mode with a MS scan range of 200-3000 m/z and MS/MS scan range of 50-3200 m/z. Data were extracted with MH+ mass range between 50 and 3200 Da and processed with Agilent Spectrum Mill MS Proteomics Workbench software packages version B.04.00 against merged database incorporating both non-redundant NCBI databases of Serpentes (taxid: 8570) and in-house transcripts database as previously described [26, 27]. Carbamidomethylation was specified as a fixed modification and oxidized methionine as a variable modification. The identified proteins or peptides were validated with the following filters: protein score > 20, peptide score > 10 and scored peak intensity (SPI) > 70%. Identified proteins were filtered to achieve false discovery rate (FDR) < 1% for the peptide-spectrum matches. The abundance of individual venom toxin was estimated based on its mean spectral intensity (MSI) relative to the total MSI of all proteins identified through the in-solution mass spectrometry [28]. The relative abundance of individual venom protein within each chromatographic step was estimated based on the mean spectral intensity (MSI) of its peptides relative to the total MSI of all proteins detected in the fraction [29].

###  Immunoprofiling of *D. siamensis* Venoms 

This assay was performed for assessing the immunorecognition of antivenoms (DsMAV-Thai and SABU) against the eluted HPLC protein fractions with an indirect enzyme-linked immunosorbent assay (ELISA) protocol as previously described [30]. In brief, immunoplate wells were precoated overnight with 10 ng of HPLC eluted fractions of both Ds-Thailand and Ds-Indonesia. The plate was then flicked dry and rinsed four times with phosphate-buffered saline containing 0.5% Tween 20 (PBST). The dilution of the antivenoms, DsMAV-Thai and SABU, was standardized at an optimum concentration (1:2700) [22] based on a working concentration of 20 mg/mL each. A hundred microliters of antivenom was added to each antigen-coated well, followed by incubation for 1 hour at room temperature. 

Upon washing the plates four times with PBST, 100 µL of appropriately diluted horseradish peroxidase-conjugated-anti-horse-IgG in PBST was added to the well and incubated for another hour at room temperature. The excess components were removed by washing four times with PBST. Subsequently, 100 µL of freshly prepared substrate solution (0.5 mg/mL o-phenylenediamine and 0.003% hydrogen peroxide in 0.1 M citrate-phosphate buffer, pH 5.0) were added to each well. The enzymatic reaction was allowed to take place in the dark for 30 min at room temperature. The reaction was terminated by adding 50 µL of 12.5% sulfuric acid, and the absorbance at 492 nm was read against blank using Tecan Infinite M1000 Pro plate reader. Immunoreactivity was expressed as percentage of relative absorbance between two comparing antivenoms toward the HPLC fractions of Ds-Thailand and Ds-Indonesia. The values were means of triplicates ± standard errors of mean (S.E.M).

## Results

### Decomplexing Profile of *D. siamensis* Venom from Thailand and Indonesia

**Figure 1.**
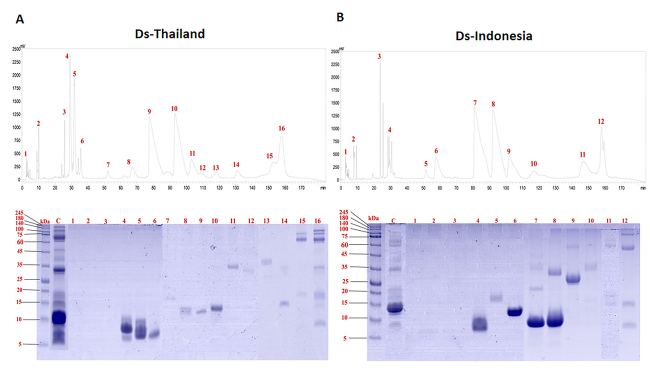
Protein decomplexation of *Daboia siamensis* venom from **(A)** Thailand and **(B)** Indonesia. Upper panel: C18 reverse-phase high-performance liquid chromatography (HPLC) profile of the venom. Lower panel: 15% sodium dodecyl sulfate-polyacrylamide gel electrophoresis (SDS-PAGE) of the protein fractions, under reducing conditions. Lane C: whole venom.

The reverse-phase HPLC of *D. siamensis* venoms from Thailand and Indonesia yielded 16 fractions (Ds-Thailand) (Fig. 1A) and 12 fractions (Ds-Indonesia) (Fig. 1B), respectively. SDS-PAGE revealed the presence of proteins in fraction 4 (corresponding to 30 min elution time) onward. The fractions eluted in the initial 100 min for both venoms mainly contained low molecular weight proteins (~7-15 kDa) and most of the high molecular weight proteins (> 30 kDa) were eluted between 120 min to 170 min. Notably, the Indonesian *D. siamensis* venom HPLC profile revealed the presence of two unique fractions i.e. fraction 6 (eluted at 60 min) and fraction 11 (eluted at 145 min), with fraction 6 containing a corresponding ~10 kDa protein, and fraction 11 containing proteins of ~15-20 kDa and ~60-70 kDa.

**Proteomic Analysis of Thai and Indonesian *Daboia siamensis* Venoms**

The proteins eluted through HPLC of the Thai and Indonesian *D. siamensis* venoms were identified and sorted according to the relative protein abundance (%) and the respective fraction (Tables 1 and 2). The peptide sequences and mass spectrometry data (including ion mass/charge data of peptide) are available in Additional files 1 and 2.

**Figure 2.**
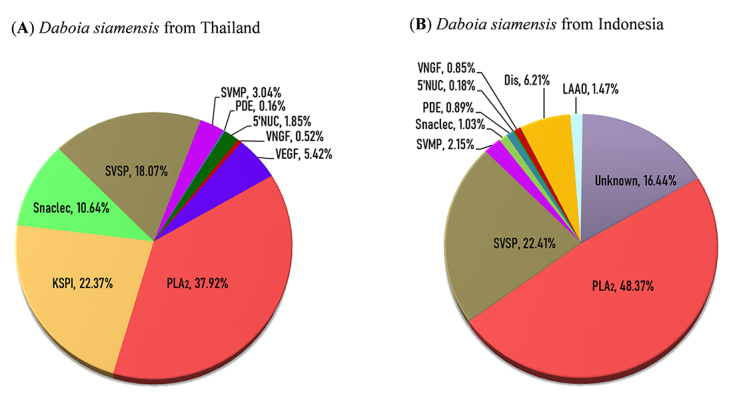
Venom proteomes of *Daboia siamensis* from **(A)** Thailand and **(B)** Indonesia. KSPI: Kunitz-type serine protease inhibitor; PLA_2_: phospholipase A_2_; snaclec: snake venom C-type lectin/lectin-like protein; SVSP: snake venom serine protease; SVMP: snake venom metalloproteinase; LAAO: L-amino acid oxidase; VEGF: snake venom vascular endothelial growth factor; VNGF: snake venom nerve growth factor; 5′NUC: 5′-nucleotidase; PDE: phosphodiesterase; Dis: disintegrin.

**Table 1.** Venom proteins of *Daboia siamensis* (Thailand) assigned by fractions of reverse-phase HPLC. Data derived from LC-MS/MS analysis of the tryptic peptides

Frac.	Protein score	Protein name*	Accession number^#^	Species	Relative abundance (%)
**4**					**9.83%**
	192.8	Kunitz-type serine protease inhibitor C1	A8Y7N4	*D. siamensis*	7.37
	52.94	Kunitz-type serine protease inhibitor C6	A8Y7N9	*D. siamensis*	0.54
	38.91	Kunitz-type serine protease inhibitor B6	A8Y7P6	*D. siamensis*	1.92
**5**					**9.14%**
	67.75	Kunitz-type serine protease inhibitor C1	A8Y7N4	*D. siamensis*	0.65
	85.22	Kunitz-type serine protease inhibitor C2	A8Y7N5	*D. siamensis*	0.54
	48.9	Kunitz-type serine protease inhibitor C4	A8Y7N7	*D. siamensis*	2.59
	30.32	Kunitz-type serine protease inhibitor C6	A8Y7N9	*D. siamensis*	4.29
	85.09	Kunitz-type serine protease inhibitor DrKIn-II	H6VC06	*D. russelii*	0.25
	72.37	Kunitz-type serine protease inhibitor 2	P00990	*D. siamensis*	0.82
**6**					**3.14%**
	63.41	Kunitz-type serine protease inhibitor C1	A8Y7N4	*D. siamensis*	0.20
	70.78	Kunitz-type serine protease inhibitor C2	A8Y7N5	*D. siamensis*	0.08
	52.04	Kunitz-type serine protease inhibitor C4	A8Y7N7	*D. siamensis*	1.10
	29.22	Kunitz-type serine protease inhibitor C6	A8Y7N9	*D. siamensis*	0.20
	31.54	Kunitz-type serine protease inhibitor B5	A8Y7P5	*D. siamensis*	0.66
	39.97	Kunitz-type serine protease inhibitor DrKIn-II	H6VC06	*D. russelii*	0.07
	63.82	Kunitz-type serine protease inhibitor 2	P00990	*D. siamensis*	0.59
	34.41	Coagulation factor X-activating enzyme heavy chain	Q7LZ61	*D. siamensis*	0.23
**7**					**0.92%**
	44.81	Kunitz-type serine protease inhibitor C2	A8Y7N5	*D. siamensis*	0.07
	31.75	Kunitz-type serine protease inhibitor DrKIn-II	H6VC06	*D. russelii*	0.04
	47.6	Snake venom vascular endothelial growth factor toxin VR-1	P67861	*D. russelii*	0.39
	79.52	Venom nerve growth factor 1	V9I1K1	*D. russelii*	0.43
**8**					**3.19%**
	46.88	Kunitz-type serine protease inhibitor C1	A8Y7N4	*D. siamensis*	0.10
	45.33	Kunitz-type serine protease inhibitor C2	A8Y7N5	*D. siamensis*	0.05
	36.24	Kunitz-type serine protease inhibitor C4	A8Y7N7	*D. siamensis*	0.05
	56.41	Phospholipase A_2_	B2YHV5	*D. siamensis*	0.24
	53.05	Basic phospholipase A_2_ beta-bungarotoxin A1 chain	P00617	*B. multicinctus*	0.05
	50.79	Basic phospholipase A_2_ beta-bungarotoxin A1 chain	P00617	*B. multicinctus*	0.05
	35.5	Snake venom vascular endothelial growth factor toxin VR-1'	P0DL42	*D. siamensis*	2.64
**9**					**26.23%**
	174.64	Basic phospholipase A_2_	B3RFI7	*D. r. limitis*	12.63
	43.61	Kunitz-type serine protease inhibitor C1	A8Y7N4	*D. siamensis*	0.10
	21.86	Kunitz-type serine protease inhibitor C2	A8Y7N5	*D. siamensis*	0.06
	65.41	Phospholipase A_2_	B2YHV5	*D. siamensis*	0.88
	52.49	Beta-fibrinogenase	E0Y419	*M. lebetina*	1.38
	52.09	Serine protease VLSP-3	E0Y420	*M. lebetina*	4.22
	32.37	Beta-fibrinogenase-like	E5L0E4	*D. siamensis*	3.10
	24.5	Snake venom vascular endothelial growth factor toxin VR-1	P67861	*D. russelii*	2.00
	56.87	Venom serine proteinase-like protein 2	Q9PT40	*M. lebetina*	1.75
	35.87	Venom nerve growth factor 1	V9I1K1	*D. russelii*	0.09
**10**					**23.34%**
	36.23	Beta-fibrinogenase-like	E5L0E4	*D. siamensis*	0.12
	133.44	Acidic phospholipase A_2_ RV-7	P31100	*D. siamensis*	11.00
	195.54	Acidic phospholipase A2 RV-7	P31100	*D. siamensis*	9.52
	30.91	Phospholipase A_2_ 1	P86529	*D. russelii*	2.69
**11**					**4.17%**
	94.05	Alpha-fibrinogenase-like	E5L0E3	*D. siamensis*	0.25
	291.13	Factor V activator RVV-V gamma	P18965	*D. siamensis*	1.95
	274.2	Factor V activator RVV-V gamma	P18965	*D. siamensis*	1.86
	58.76	Acidic phospholipase A_2_ RV-7	P31100	*D. siamensis*	0.12
**12**					**0.54%**
	37.01	Beta-fibrinogenase	E0Y419	*M. lebetina*	0.02
	29.97	Serine protease VLSP-3	E0Y420	*M. lebetina*	0.02
	72.73	Alpha-fibrinogenase-like	E5L0E3	*D. siamensis*	0.03
	244.98	RVV-V gamma-like protein precursor	P18965	*D. siamensis*	0.10
	181.46	Factor V activator RVV-V gamma	P18965	*D. siamensis*	0.14
	84.44	Acidic phospholipase A_2_ RV-7	P31100	*D. siamensis*	0.09
	37.42	Venom serine proteinase-like protein 2	Q9PT40	*M. lebetina*	0.01
	78.54	Serine beta-fibrinogenase	CL2958.Contig2_DrSL	*D. russelii*	0.13
**13**					**1.17%**
	42	Kunitz-type serine protease inhibitor C1	A8Y7N4	*D. siamensis*	0.002
	25.6	Kunitz-type serine protease inhibitor B6	A8Y7P6	*D. siamensis*	0.01
	34.12	Alpha-fibrinogenase-like	E5L0E3	*D. siamensis*	0.31
	153.15	Factor V activator RVV-V gamma	P18965	*D. siamensis*	0.28
	133.87	Factor V activator RVV-V gamma	P18965	*D. siamensis*	0.23
	75.58	Acidic phospholipase A_2_ RV-7	P31100	*D. siamensis*	0.03
	46.83	Factor V activator RVV-V gamma	Unigene26743_DrSL	*D. russelii*	0.32
**14**					**1.48%**
	37.58	Serine protease VLSP-3	E0Y420	*M. lebetina*	0.03
	192.24	Alpha-fibrinogenase-like	E5L0E3	*D. siamensis*	0.29
	32.79	P31 alpha subunit	K9JBU9	*D. siamensis*	0.02
	146.91	Factor V activator RVV-V gamma	P18965	*D. siamensis*	0.18
	134.03	Factor V activator RVV-V gamma	P18965	*D. siamensis*	0.13
	86.12	Acidic phospholipase A_2_ RV-7	P31100	*D. siamensis*	0.12
	21.67	Snake venom vascular endothelial growth factor toxin VR-1	P67861	*D. russelii*	0.07
	51.59	Basic phospholipase A_2_ RV-4	Q02471	*D. siamensis*	0.10
	48.78	Snaclec 4	Q4PRC9	*D. siamensis*	0.02
	68.15	Serine beta-fibrinogenase	CL2958.Contig2_DrSL	*D. russelii*	0.16
	115.48	RVV-V gamma-like protein precursor	CL310.Contig22_NnSL	*N. naja*	0.23
	41.42	Serine protease VLSP-1	CL310.Contig24_NnSL	*N. naja*	0.13
**15**					**5.17%**
	25.78	Alpha-fibrinogenase-like	E5L0E3	*D. siamensis*	0.02
	28.57	P31 beta subunit	K9JBV3	*D. r. limitis*	0.35
	32.42	P31 beta subunit	K9JDF6	*D. siamensis*	0.23
	85.94	Factor V activator RVV-V gamma	P18965	*D. siamensis*	0.13
	96.25	Acidic phospholipase A_2_ RV-7	P31100	*D. siamensis*	0.33
	58.85	Snaclec coagulation factor X-activating enzyme light chain 1	Q4PRD1	*D. siamensis*	0.21
	120.28	Coagulation factor X-activating enzyme heavy chain	Q7LZ61	*D. siamensis*	0.12
	31.13	P31 alpha subunit	CL1101.Contig2_DrSL	*D. russelii*	3.54
	41.97	P68 alpha subunit	CL2900.Contig2_NnSL	*N. naja*	0.03
	56.67	Serine protease VLSP-1	CL2958.Contig2_DrSL	*D. russelii*	0.07
	33.15	Serine protease VLSP-1	CL310.Contig24_NnSL	*N. naja*	0.13
**16**					**11.68%**
	82.86	RVV-X light chain 1	A0A2H4Z2X7	*D. siamensis*	1.35
	35.54	Alpha-fibrinogenase-like	E5L0E3	*D. siamensis*	0.04
	36.29	P31 alpha subunit	K9JBU9	*D. siamensis*	0.17
	41.85	P68 alpha subunit	K9JBV0	*D. siamensis*	1.01
	95.46	Factor X activator light chain 2	K9JDJ1	*D. siamensis*	0.25
	21.91	Snake venom vascular endothelial growth factor toxin VR-1'	P0DL42	*D. siamensis*	0.32
	54.47	Factor V activator RVV-V gamma	P18965	*D. siamensis*	0.11
	43.48	Acidic phospholipase A_2_ RV-7	P31100	*D. siamensis*	0.08
	72.99	Snaclec 3	Q4PRD0	*D. siamensis*	0.32
	129.18	Snaclec coagulation factor X-activating enzyme light chain 1	Q4PRD1	*D. siamensis*	1.17
	48.29	Snaclec coagulation factor X-activating enzyme light chain 2	Q4PRD2	*D. siamensis*	0.38
	193.33	Coagulation factor X-activating enzyme heavy chain	Q7LZ61	*D. siamensis*	1.32
	172.13	Coagulation factor X-activating enzyme heavy chain	Q7LZ61	*D. siamensis*	1.37
	115.48	5'-nucleotidase	U3T7C6	*O. okinavensis*	0.54
	118.19	Phosphodiesterase	U3TBJ5	*O. okinavensis*	0.08
	229.87	5'-nucleotidase	W8EFS0	*M. ebetina*	0.39
	58.32	Factor X activator light chain 1	CL290.Contig28_NnSL	*N. naja*	1.58
	199.35	Snake venom 5'-nucleotidase OS	CL2941.Contig2_EcSL	*E. carinatus*	0.53
	49.82	Serine beta-fibrinogenase	CL2958.Contig2_DrSL	*D. russelii*	0.05
	71.65	RVV-V gamma-like protein precursor	CL310.Contig22_NnSL	*N. naja*	0.08
	29.49	Serine protease VLSP-1	CL310.Contig24_NnSL	*N. naja*	0.05
	288.98	Snake venom 5'-nucleotidase	CL3322.Contig2_DrSL	*D. russelii*	0.39
	297.24	Phosphodiesterase 1	CL3655.Contig2_DrSL	*D. russelii*	0.08

Protein names (*) and accession numbers (^#^) were identified based on homology search of tryptic peptides against protein database used in the study.

**Table 2.** Venom proteins of *Daboia siamensis* (Indonesia) assigned by fractions of reverse-phase HPLC. Data derived from LC-MS/MS analysis of the tryptic peptides

Frac.	Protein score	Protein name*	Accession number^#^	Species	Relative abundance (%)
**4**					**6.21%**
	20.01	Disintegrin jerdostatin	Q7ZZM2	*P. jerdonii*	6.21
5					0.85%
	66.21	Venom nerve growth factor 1	V9I1K1	*D. russelii*	0.85
**6**		(Unidentified)			**7.47%**
**7**					**32.61%**
	119.66	Basic phospholipase A_2_	B3RFI7	*D. r. limitis*	21.25
	56.61	Venom serine proteinase-like protein 2	Q9PT40	*M. lebetina*	11.37
**8**					**31.47%**
	76.93	Acidic phospholipase A_2_ Drk-a1	A8CG86	*D. russelii*	4.95
	50.37	Basic phospholipase A_2_	B3RFI7	*D. r. limitis*	0.17
	35.19	Serine protease VLSP-3	E0Y420	*M. lebetina*	0.13
	56.33	Alpha-fibrinogenase-like	E5L0E3	*D. siamensis*	0.12
	38.5	Beta-fibrinogenase-like	E5L0E4	*D. siamensis*	0.18
	224.61	Factor V activator RVV-V gamma	P18965	*D. siamensis*	2.03
	182.85	Acidic phospholipase A_2_ RV-7	P31100	*D. siamensis*	16.83
	32.28	Phospholipase A_2_ 1	P86529	*D. russelii*	4.89
	217.36	RVV-V gamma-like protein precursor Daboia-russelii	CL29580.Contig2_DrSL	*D. russelii*	2.18
**9**					**9.06%**
	21.24	Serine protease VLSP-3	E0Y420	*M. lebetina*	4.53
	92.33	Alpha-fibrinogenase-like	E5L0E3	*D. siamensis*	0.35
	168.8	Factor V activator RVV-V gamma	P18965	*D. siamensis*	1.48
	171.75	Factor V activator RVV-V gamma	P18965	*D. siamensis*	0.92
	36	Acidic phospholipase A_2_ RV-7	P31100	*D. siamensis*	0.24
	74.09	Factor V activator RVV-V gamma	Unigene26743_EsM	*E. schistosa*	1.54
**10**					**2.78%**
	45.57	Factor V activator RVV-V gamma	P18965	*D. siamensis*	0.08
	45.95	Acidic phospholipase A_2_ RV-7	P31100	*D. siamensis*	0.05
	74.97	Snaclec coagulation factor X-activating enzyme light chain 1	Q4PRD1	*D. siamensis*	0.21
	43.43	Snaclec coagulation factor X-activating enzyme light chain 2	Q4PRD2	*D. siamensis*	0.29
	40.23	Factor X activator light chain 2	T1P647	*D. russelii*	0.32
	50.26	Serine protease VLSP-1	CL29580.Contig2_DrSL	*D. russelii*	1.79
	64.24	RVV-V gamma-like protein precursor	CL3102.Contig2_NnSL	*N. naja*	0.04
**11**		(Unidentified)			**8.97%**
12					4.90%
	47.46	Coagulation factor X-activating enzyme heavy chain	Q7LZ61	*D. siamensis*	1.83
	142.43	L-amino-acid oxidase	G8XQX1	*D. russelii*	0.53
	96.77	P68 alpha subunit	K9JDF2	*D. r. limitis*	0.44
	71.25	L-amino-acid oxidase	P0C2D7	*V. berus berus*	0.94
	53.81	Snaclec 3	Q4PRD0	*D. siamensis*	0.09
	96.88	Phosphodiesterase	U3TBJ5	*O. okinavensis*	0.60
	113.5	Snake venom 5'-nucleotidase	CL3322.Contig21_DrSL	*D. russelii*	0.18
	305.76	Phosphodiesterase 1	CL3655.Contig2_DrSL	*D. russelii*	0.29

Protein names (*) and accession numbers (#) were identified based on homology search of tryptic peptides against protein database used in the study.

The proteomic analysis using nano-ESI LC-MS/MS identified a total of 47 non-redundant proteins belonging to nine protein families in Ds-Thailand venom and 25 proteins pertaining to nine protein families in the Ds-Indonesia venom (Table 3). The protein families co-expressed in both venoms are phospholipase A_2_ (PLA_2_), snake venom C-type lectin/lectin-like protein (snaclec), snake venom serine protease (SVSP), snake venom metalloproteinase (SVMP), snake venom nerve growth factor (VNGF), 5’-nucleotidase (5’NUC) and phosphodiesterase (PDE). 

**Table 3.** Comparison of protein families, toxin subtypes and relative abundances in the venoms of *Daboia siamensis* from Thailand and Indonesia

Protein family and subtype	Accession number	Relative abundance (% total venom protein)	
		***D. siamensis* (Thailand)**	***D. siamensis* (Indonesia)**
**Kunitz-type serine protease inhibitor (KSPI)**		**22.38**	-
Kunitz-type serine protease inhibitor 2	P00990	1.4	-
Kunitz-type serine protease inhibitor B5	A8Y7P5	0.66	-
Kunitz-type serine protease inhibitor B6	A8Y7P6	1.93	-
Kunitz-type serine protease inhibitor C1	A8Y7N4	8.43	-
Kunitz-type serine protease inhibitor C2	A8Y7N5	0.81	-
Kunitz-type serine protease inhibitor C4	A8Y7N7	3.75	-
Kunitz-type serine protease inhibitor C6	A8Y7N9	5.04	-
Kunitz-type serine protease inhibitor DrKIn-II	H6VC06	0.36	-
**Phospholipase A_2_ (PLA_2_)**		37.92	**48.37**
Acidic phospholipase A_2_ Drk-a1	A8CG86	-	4.95
Acidic phospholipase A_2_ RV-7	P31100	21.27	17.12
Basic phospholipase A_2_	B3RFI7	12.63	21.41
Basic phospholipase A2 beta-bungarotoxin A1 chain	P00617	0.1	-
Basic phospholipase A_2_ RV-4	Q02471	0.1	-
Phospholipase A2	B2YHV5	1.13	-
Phospholipase A2 1	P86529	2.69	4.89
**Snake venom C-type lectin/lectin-like protein (snaclec)**	**10.63**	**1.03**	
Factor X activator light chain 1	CL290.Contig28_NnSL	1.58	-
Factor X activator light chain 2	K9JDJ1	0.25	-
P31 alpha subunit	K9JBU9	0.19	-
P31 alpha subunit	CL1101.Contig2_DrSL	3.54	-
P31 beta subunit	K9JBV3	0.35	-
P31 beta subunit	K9JDF6	0.23	-
P68 alpha subunit	K9JBV0	1.01	-
P68 alpha subunit	K9JDF2	-	0.44
P68 alpha subunit	CL2900.Contig2_NnSL	0.03	-
RVV-X light chain 1	A0A2H4Z2X7	1.35	-
Snaclec 3	Q4PRD0	0.32	0.09
Snaclec 4	Q4PRC9	0.02	
Snaclec coagulation factor X-activating enzyme light chain 1	Q4PRD1	1.38	0.21
Snaclec coagulation factor X-activating enzyme light chain 2	Q4PRD2	0.38	0.29
**Snake venom serine protease (SVSP)**		**18.07**	**22.41**
Alpha-fibrinogenase-like	E5L0E3	0.95	0.47
Beta-fibrinogenase	E0Y419	1.41	-
Beta-fibrinogenase-like	E5L0E4	3.22	0.18
Factor V activator RVV-V gamma	P18965	5.11	4.5
Factor V activator RVV-V gamma	Unigene26743_DrSL	0.32	-
Factor V activator RVV-V gamma	Unigene26743_EsM	-	1.54
RVV-V gamma-like protein precursor	CL3102.Contig2_NnSL	0.31	0.04
Serine beta-fibrinogenase	CL2958.Contig2_DrSL	0.4	3.97
Serine protease VLSP-1	CL310.Contig24_NnSL	0.32	-
Serine protease VLSP-3	E0Y420	4.27	0.34
Venom serine proteinase-like protein 2	Q9PT40	1.76	11.37
**Snake venom metalloproteinase (SVMP)**		**3.04**	**2.15**
Coagulation factor X-activating enzyme heavy chain	Q7LZ61	3.04	1.83
Factor X light activator chain 2	T1P647	-	0.32
**L-amino acid oxidase (LAAO)**		-	**1.47**
L-amino-acid oxidase	G8XQX1	-	0.53
L-amino-acid oxidase	P0C2D7	-	0.94
**Snake venom vascular endothelial growth factor (svVEGF)**	**5.42**	
Snake venom vascular endothelial growth factor toxin VR-1	P67861	2.46	-
Snake venom vascular endothelial growth factor toxin VR-1'	P0DL42	2.96	-
**Snake venom nerve growth factor (svNGF)**		**0.52**	**0.85**
Venom nerve growth factor 1	V9I1K1	0.52	0.85
**Snake venom 5′-nucleotidase (5′NUC)**		**1.85**	**0.18**
5’-nucleotidase	U3T7C6	0.54	-
5'-nucleotidase	W8EFS0	0.39	-
Snake venom 5’-nucleotidase	CL3322.Contig1_DrSL	-	0.18
Snake venom 5'-nucleotidase	CL3322.Contig2_DrSL	0.39	-
Snake venom 5'-nucleotidase OS	CL2941.Contig2_EcSL	0.53	-
**Phosphodiesterase (PDE)**		**0.16**	**0.89**
Phosphodiesterase	U3TBJ5	0.08	0.6
Phosphodiesterase 1	CL3655.contig2_DrSL	0.08	0.29
**Disintegrin**		-	**6.21**
Disintegrin jerdostatin	Q7ZZM2	-	6.21
**Unidentified proteins**	-	-	**16.44**

The two most abundant protein families (in terms of total venom proteins) in Ds-Thailand venom are PLA_2_ and KSPI. In Ds-Indonesia venom, the most abundantly present proteins are PLA_2_ and SVSP (Fig. 2). The most abundant protein family in the venoms of both species is PLA_2_. A total of six PLA_2_ forms were detected in Ds-Thailand venom, and four PLA_2_ forms were identified in Ds-Indonesia venom. The distinctive PLA_2_ proteoform seen in both venoms were acidic phospholipase A_2_ RV-7 and basic phospholipase A_2_. The relative abundance of SVSP (by total venom proteins) is higher in Ds-Indonesia venom, and the proteoforms of SVSP detected also varied between the two *D. siamensis* venoms. A total of ten SVSP forms was detected in Ds-Thailand venom and eight SVSP forms were detected in Ds-Indonesia venom although the latter has a higher relative abundance by total venom proteins. Snaclec (snake venom C-type lectin/lectin- like protein) were more abundant in Ds-Thailand venom (10.63%) compared to Ds-Indonesia (1.03%). Kunitz-type serine protease inhibitors (KSPI, 22.38%) and snake venom vascular endothelial growth factor (VEGF, 5.42%) were only detected in Ds-Thailand venom, whereas L-amino acid oxidase (LAAO, 1.47%) and disintegrin (Dis, 6.21%) were only detected in Ds-Indonesia venom. Besides, both venoms shared three minor protein families at a low abundance (< 2% of total venom proteins) i.e. VNGF, PDE and 5’NUC (Fig. 2). The fractions 6 and 11 of Ds-Indonesia venom, however, were unidentified by the routine protease-digestion and LC-MS/MS approach.

### Immunoprofiling of Thai and Indonesian *D. siamensis* venom proteins

**Figure 3.**
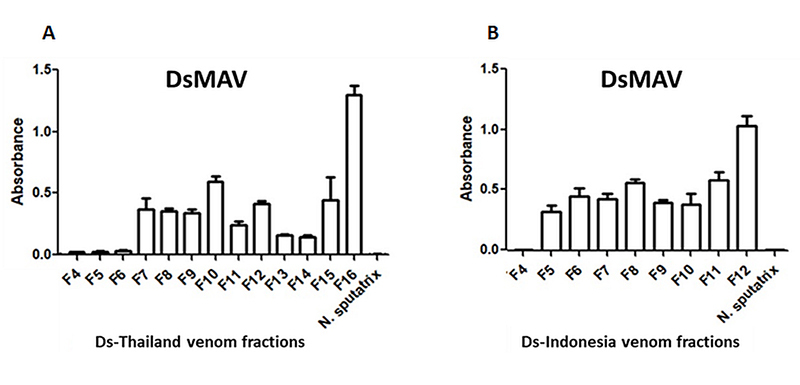
Immunoreactivity of Thai *Daboia siamensis* monovalent antivenom (DsMAV-Thailand) toward HPLC venom protein fractions of *D. siamensis* from **(A)** Thailand and **(B)** Indonesia. *Naja sputatrix* venom was used as negative control. Absorbance values were obtained by indirect ELISA and expressed as mean ± S.E.M. from three independent experiments.

Figure 3 illustrates the immunological binding activities of DsMAV-Thailand and SABU toward the fractionated venom proteins of the Thai and Indonesian *D. siamensis*. DsMAV-Thailand showed a low immunological binding activity toward the proteins in fractions F4 to F6 of Ds-Thailand, with absorbance detected below 0.1. The immunological binding activity of the antivenom was apparently higher in fractions F7 to F16, with absorbance detected in the range of 0.2 to 1.5 (Fig. 3A). The highest immunological binding activity of DsMAV-Thailand was noted toward the last fraction F16, which contained primarily high molecular weight proteins. 

**Figure 4.**
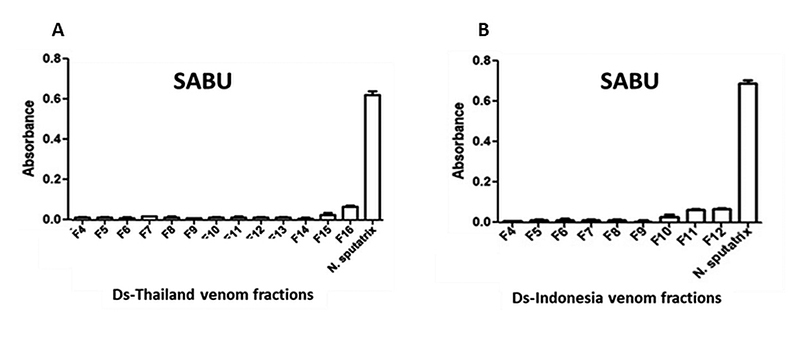
Immunoreactivity of Indonesian Serum Anti Bisa Ular (SABU) toward HPLC venom protein fractions of *D. siamensis* from **(A)** Thailand and **(B)** Indonesia. *Naja sputatrix* venom was used as positive control. Absorbance values were obtained by indirect ELISA and expressed as mean ± S.E.M. from three independent experiments.

The Thai antivenom exhibited a similar binding profile toward the protein fractions of Ds-Indonesia venom (Fig. 3B), where the protein in fraction F4 was weakly bound while the immunoreactivity increased from fractions F5 to F12 (0.2−1.5 absorbance). The Indonesian antivenom SABU, on the other hand, was extremely poor in the immunorecognition of venom proteins from both Russell’s viper populations (Fig. 4A and Fig. 4B), with absorbance detected below 0.1 for all the protein fractions. 

## Discussion

Snake venoms may present intraspecific variation in relation to protein composition due to geographical factors [2]. The knowledge of the geographical variation of Russell’s viper venom from Thailand and Indonesia is essential to understand the potential variable toxicity and dissimilar antigenicity of the venom proteins. The present work successfully unveiled the venom proteomes of two distant populations of *D. siamensis* using a comparative analytical approach. 

The present findings reveal that most protein families - i.e. PLA_2_, snaclec, SVSP, SVMP, PDE, 5’NUC and VNGF - were well conserved between the two geographical populations despite their wide disjunctive distribution. Nevertheless, the protein profiles of the two venoms were not identical, because variations in the presence of KSPI, LAAO and disintegrin were observed, as well as the expression level of snaclec. In addition, the unidentified proteins in two unique fractions from the Indonesian venom sample also implies the presence of novel proteins with sequence variants that are divergent from the Thai sample. Admittedly, the limitation could be possibly due to unavailable sequences homologous to the proteins that are novel and previously uncharacterized. These proteins deserve further investigation including use of sequence databases from genome and venom-gland transcriptome specific to *D. siamensis* of Indonesian origin. 

To further understand the protein composition and variations within the Russell’s viper complex, the venom proteomics reported for both the western (*D. russelii*) and eastern (*D. siamensis*) lineages were compared in Table 4. The PLA_2_ family is found to be, consistently, the most abundant in both the eastern and western Russell’s viper venoms. PLA_2_ in snake venom plays a major role in a wide range of pharmacological activities, including neurotoxicity, edema formation, platelet aggregation and anticoagulant action [31]. In addition to prey digestion, PLA_2_ in a single snake can be represented by several isoenzymes that exhibit diverse pharmacological effects [32, 33]. Comparatively, the high abundance of PLA_2_ in both *D. siamensis* from Thailand (37.92%) and Indonesia (48.37%) is greater than that reported for *D. siamensis* from Guangxi, China (22.2%) and Taiwan (24.5%) [18]. The high abundance of PLA_2_ is also comparable to that described for Russell’s viper from Sri Lanka (35%), western India (32.5%) and Pakistan, where PLA_2_ is lower in captive snakes (32.8%) than in wild-caught specimens (63.8%) [5-8]. 

In addition, the presence of variable PLA_2_ forms was also qualitatively detected in the proteomes of *D*. *siamensis* from Myanmar and *D. russelii* from southern India [19, 34]. Both acidic and basic PLA_2_ constitute the protein family in all Russell’s viper venoms at varying ratios. In Ds-Thailand and Ds-Indonesia venoms, one of the major PLA_2_ isoforms is homologous to the acidic phospholipase A_2_ RV-7 (21.3%). It has been reported that PLA_2_ RV-4 and RV-7 is a heterodimeric complex that could induce presynaptic neurotoxicity [18, 35]. The level of PLA_2_ RV-4 detected in Ds-Thailand and Ds-Indonesia is, however, considerably low and this may reflect the lack of neurotoxicity in Thai and Indonesian Russell’s viper envenoming [36]. Neurotoxicity is a common clinical feature observed in victims of *D. russelii* bites in Sri Lanka and in some parts of India [11, 36-38] but not commonly observed in envenomation of Eastern Russell’s viper in Southeast Asia [36], Taiwan or China, where coagulopathy appears to be the major clinical manifestation [14, 39]. 

**Table 4.** Venom proteomic profiles of Russell’s viper (*Daboia siamensis* and *Daboia russelii*). Percentage values indicate the relative abundances of the proteins (by total venom protein)

Species and locale	*D. siamensis* (Thailand - pooled sample)	*D. siamensis* (Java, Indonesia - pooled sample)	*D.* siamensis (Insular Taiwan - pooled sample)	*D. siamensis* (Taiwan - captive - polled sample)	*D. siamensis* (Guangxi, China - n ?#61619; 10 adults)	*D. siamensis* (Myanmar - pooled sample)	*D. russelii* (Western, India - n = 4 adults)	*D. russelii* (Tamil Nadu, India - pooled sample)	*D. russelii* (Pakistan - wild - n ?#61619;10 adults)	*D. russelii* (Pakistan - captive - n = 3 adults)	*D. russelii* (Sri Lanka - n ?#61619;5 adults)
LD_50_ (µg/g mouse)	0.34 (0.30-0.38) (i.v.)	0.22 (0.20-0.24) (i.v.)	0.09 (0.06-0.14) (i.v.)	0.47 (i.p.)	0.18 (0.12-0.27) (i.v.)	NA	NA	NA	0.19 (0.17-0.25) (i.v.)	NA	NA
Reference	Current study	Current study	[18]	[17]	[18]	[19]	[6]	[34]	[5]	[7]	[8]
Method	C18 rpHPLC, in-solution tryptic digestion, LC-MS/MS	C18 rpHPLC, in-solution tryptic digestion, LC-MS/MS	In-gel tryptic digestion, LC-MS/MS	C18 rpHPLC, in-gel tryptic digestion, LC-MS/MS	In-gel tryptic digestion, LC-MS/MS	2D gel, tryptic digestion	Gel filtration chromatography, in-solution tryptic digestion, LC-MS/MS	C18 rpHPLC, in-gel tryptic digestion, LC-MS/MS	Gel filtration chromatography, in-solution tryptic digestion, LC-MS/MS	C18 rpHPLC, in-solution tryptic digestion, LC-MS/MS	In-gel tryptic digestion, LC-MS/MS
KSPI	22.38	-	28.20	9.4	23.17	-	12.50	P	16.00	28.4	4.70
PLA_2_	37.92	48.37	24.47	47.5	22.18	P	32.50	P	63.80	32.8	35.00
*Acidic*	*25.09*	*26.96*	*10.87*	*21.2*	*14.76*	*P*	*P*	*P*	*21.51*	*P*	*34.7*
*Basic*	*12.83*	*21.41*	*13.61*	*26.3*	*7.42*	*P*	*P*	*P*	*42.30*	*P*	*0.3*
*Neutral*	-	-	-	-	-	-	-	-	-	-	-
Snaclec	10.64	1.03	16.52	5.2	16.89	P	1.80	P	1.30	6.4	22.40
SVSP	18.07	22.41	17.5	19.1	13.61	P	8.00	P	5.50	3.2	16.00
SVMP	3.04	2.15	5.85	9.2	8.92	P	24.80	P	2.50	21.8	6.90
LAAO	-	1.47	-	-	5. 95	P	0.30	P	0.80	0.6	5.20
svNGF	0.52	0.85	2.13	0.3	2.11	-	4.80	P	1.10	0.4	3.42
svVEGF	5.42	-	4.84	7.2	4.79	P	1.80	P	4.30	1.5	-
5’NUC	1.85	0.18	0.17	-	0.82	-	0.40	P	0.10	0.5	3.00
CRiSP	-	-	-	0.1	0.95	-	6.80	P	1.30	2.6	2.00
PDE	0.16	0.89	0.31	0.9	0.25	-	1.40	P	2.50	0.6	1.30
Disintegrin	-	6.21	-	-	-	-	4.90	P	P	0.4	-
Aminopeptidase		-	0.15	-	0.35	-	-	-	-	-	-
PLB	-	-	-	-	-	-	0.10	-	-	-	-
HLP	-	-	-	-	-	-	-	P	-	-	-
Unknown	-	16.44	-	-	-	-	-	-	-	-	-

*D. siamensis*: *Daboia siamensis*; *D. russelii*: *Daboia russelii*; i.v. LD50: intravenous median lethal dose; rpHPLC: reverse-phase high performance liquid chromatography; LC-MS/MS: liquid chromatography-tandem mass spectrometry; KSPI: Kunitz-serine protease inhibitor; PLA_2_: phospholipase A_2_; Snaclec: snake venom C-type lectin/lectin-like protein; SVSP: snake venom serine protease; SVMP: snake venom metalloproteinase; LAAO: L-amino acid oxidase; svNGF: snake venom nerve growth factor; svVEGF: snake venom vascular endothelial growth factor; 5’NUC: snake venom 5’ nucleotidase; CRiSP: Cysteine-rich secretory protein; PDE: Phosphodiesterase; PLB: phospholipase B; HLP: hypothetical like protein; NA: not available; P: present (qualitatively detected and reported).

Earlier, Mukherjee et al. [40] reported the characterization of Rusvikunin (a KSPI) from *D. russelii* venom, which has anticoagulant and antiplasmin activities, indicating that the protease inhibitor could contribute to coagulopathic effect of the venom. Venom proteomics of Russell’s viper from various locations revealed that the presence of KSPI is highly variable, from non-detectable to 29% of total venom proteins (Table 4). In the present study, KSPI was only detected in Ds-Thailand venom (22.4%), with a relative protein abundance that is similar to that reported in the comparative venom proteomes of *D. siamensis* from Taiwan (28.2%) and China (Guangxi) (23.2%) [18], as well as to captive *D. russelii* from Pakistan (28.4%) [7]. This indicates that KSPI may play a crucial role in the pathophysiology of envenomation caused by the Russell’s vipers, with potential anticoagulant activity. The KSPI abundance was reported to be much lower or negligible in the venom proteomes of Russell’s viper and from Sri Lanka [8], Myanmar [19] and Indonesia (current study). The evolutionary significance of the divergence in KSPI expression in the venom awaits further elucidation. 

*D. siamensis* envenomed patients commonly develop bleeding and coagulopathy disorder [36]. The hemotoxic activity of *D. siamensis* venom is affected by a number of toxins including SVSP, SVMP and snaclec proteins frequently detected in viperid venoms [41]. The relative abundance of snake SVSP in both Thai and Indonesian *D. siamensis* venoms is consistent with that reported for the Eastern Russell’s viper (13−23%, Table 4). SVSPs role in snake venom has notably related to consumptive coagulopathy [41-43] and in this study, several SVSP forms were detected including factor V activator RVV-V gamma which is known to selectively activate factor V in a calcium-independent manner, cleaving the Arg(1545)-Ser(1546) linkage in human factor V molecule, thus inducing human plasma coagulation [44]. On the other hand, the SVMP proteins constitute 2−3% of the total venom proteins in both *D. siamensis* venoms, comprising the factor X-activating enzyme which is a procoagulant protein that activates coagulation factor X, factor IX and protein S, thus contributing to the consumptive coagulopathic effect of the venom [45]. The low abundance of SVMP also implies that the venoms lack hemorrhagin proteins, unlike some Asiatic pit vipers whose venoms are known to cause intense dermal hemorrhage [29]. The significant presence of procoagulant SVSP and SVMP is consistent with the potent plasma-clotting effects and the absence of dermal hemorrhagic activity of both venoms from Ds-Thailand and Ds-Indonesia [22].

Meanwhile, several forms of snaclec (comprising C-type lectins and C-type lectin-like proteins of snake venom) which are non-enzymatic proteins were detected in both Ds-Thailand and Ds-Indonesia venom proteomes. Snaclecs are commonly known for platelet-modulating activity (as promoter or inhibitor of platelet aggregation), and some protein forms detected in the present study can promote calcium-dependent activation of factor X [45, 46]. The evolutionary significance of the diverged expression in snaclec between Ds-Thailand and Ds-Indonesia venom, where the latter contains a much lower ratio of snaclec, is unclear at this stage. The Ds-Indonesia venom, however, has up to 6.21% (total venom proteins) of small cysteine-rich polypeptides called disintegrins that are capable to inhibit platelet aggregation and alter hemostatic balance in envenomed victim [47], and act synergistically with SVMPs to induce hemorrhage [48]. Together, these components add to the bulk of hemotoxic proteins that contribute to coagulopathy and hemostatic derangement in Russell’s viper envenomation. 

Studies have shown that snake venom LAAO (L-amino acid oxidase) does not only exhibit antimicrobial effect, it is also a potent cytotoxic enzyme which may be involved in prey digestion [49]. In the current study, LAAO was detected only in Ds-Indonesia venom and it can be observed on SDS-PAGE under reducing conditions (as dissociated dimer around 60-70 kDa). The absence of LAAO was seen in the Russell’s viper venoms from Thailand and Taiwan [17, 18], although this enzyme is present in small amounts in the venoms of most Russell’s vipers from South Asia [7, 34], China [18] and Myanmar [9]. On the other hand, proteins that were expressed in relatively low abundance (< 1%) in the *D. siamensis* venoms such as PDE, 5’NUC and VNGF may play an ancillary role in predatory and digestive function of the venoms. VNGF can induce the release of nitric oxide and histamine [50], whereas 5’NUC and PDE can alter the extracellular levels of adenosine and other purines [51]. These activities may locally facilitate venom spread and systemically contribute to venom-induced hypotensive effect that is important for prey subjugation.

Antivenom is the only definitive treatment in snakebite envenomation, but the availability of specific, effective antivenom remains limited in many areas [52]. In Southeast Asia, the monospecific antivenom produced by the Thai Red Cross Society as the ‘antidote’ against *D. siamensis* envenoming is not available clinically in Indonesia where Russell’s viper bites are prevalent. Instead, the local community often uses the trivalent Indonesian antivenom (SABU) to treat Russell’s viper envenoming. This practice has been reported anecdotally to be ineffective (Tri Maharani, personal communication), and it is supported by our recent study which showed that SABU failed to cross-neutralize the toxicity of Indonesian *D. siamensis* venom, whereas DsMAV-Thailand was highly effective in neutralizing the Thai as well as the Indonesian *D. siamensis* venoms *in vitro* and *in vivo* [22]. 

The present study validated the clinical ineffectiveness of SABU in immunorecognizing the various proteins fractions from both Ds-Thailand and Ds-Indonesia venoms, highlighting the need for appropriate, specific antivenom to treat *D. siamensis* envenoming in Indonesia. The immunoprofiling using DsMAV-Thailand further revealed that the antivenom has comparable immunoreactivity toward the high molecular weight protein fractions in both Thai and Indonesian venom samples. The finding suggests the sharing of common protein epitopes between the two geographical venom samples. Consistently, the highest immunoreactivity was shown in fraction F16 (Ds-Thailand) and fraction F12 (Ds-Indonesia) which were predominantly SVMP, SVSPs and large proteins such as LAAO, PDE, 5’NUC. Proteins with large molecular size are usually more antigenic and hence exhibit a better immunorecognization profile as the antivenom binds more effectively to the protein antigens available [53]. The low molecular weight proteins such as KSPI and disintegrins, however, exhibit a lower immunological binding activity with antivenom presumably due to limited epitopes and antigenicity. 

## Conclusions

The present study unveiled the protein composition of *D. siamensis* venoms from Thailand and Indonesia. The protein subtypes and abundances within each family varied to some extent between the two *D. siamensis* venoms, and the variation is likely related to the geographical difference of the viper population. Immunoprofiling study revealed substantial conservation of antigenicity in the proteins of both *D. siamensis* venoms. DsMAV-Thailand was effective in immunorecognition of the protein fractions of the two venom samples, whereas SABU was virtually ineffective, consistent with the efficacy finding of the two antivenoms in neutralizing the toxicity of Indonesian *D. siamensis* venom [22]. Together, the findings support that a species-specific antivenom is required in Indonesia to treat Russell’s viper envenomation. Future studies should also aim to address the weak immunorecognition of low molecular weight proteins such as KSPI (*D. siamensis* from Thailand) and disintegrin (*D. siamensis* from Indonesia) in order to improve the antivenom potency.

### Abbreviations

 CRiSP: cysteine-rich secretory protein; *D. russelii*: *Daboia russelii*; *D. siamensis*: *Daboia siamensis*; Ds-Indonesia: *Daboia siamensis* from Indonesia; DsMAV-Thailand: Thai *D. siamensis* monovalent antivenom; Ds-Thailand: *Daboia siamensis* from Thailand; KSPI: Kunitz-serine protease inhibitor; LAAO: L-amino acid oxidase; LC-MS/MS: liquid chromatography tandem mass spectrometry; NUC: snake venom 5’ nucleotidase; PDE: phosphodiesterase; PLA_2_: phospholipase A_2_; rpHPLC: reverse-phase high performance liquid chromatography; SABU: Serum Anti Bisa Ular; snaclec: snake venom C-type lectin/lectin-like protein; SVMP: snake venom metalloproteinase; svNGF: snake venom nerve growth factor; SVSP: snake venom serine protease; svVEGF: snake venom vascular endothelial growth factor.

## Supplementary material

The following online material is available for this article:

Additional file 1. Tandem mass spectrometry analysis of Thai *Daboia siamensis* venom fractions.

Additional file 2. Tandem mass spectrometry analysis of Indonesian *Daboia siamensis* venom fractions.

## References

[B1] Wüster W (1998). The genus Daboia (Serpentes: Viperidae): Russell's
viper. Hamadryad.

[B2] Thorpe RS, Pook CE, Malhotra A (2007). Phylogeography of the Russell's viper (Daboia russelii) complex
in relation to variation in the colour pattern and symptoms of
envenoming. Herpetol J.

[B3] Mackessy SP (2010). Evolutionary trends in venom composition in the Western
Rattlesnakes (Crotalus viridis sensu lato): Toxicity vs.
tenderizers. Toxicon.

[B4] Casewell NR, Wüster W, Vonk FJ, Harrison RA, Fry BG (2013). Complex cocktails: the evolutionary novelty of
venoms. Trends Ecol Evol.

[B5] Faisal T, Tan KY, Sim SM, Quraishi N, Tan NH, Tan CH (2018). Proteomics, functional characterization and antivenom
neutralization of the venom of Pakistani Russell's viper (Daboia russelii)
from the wild. J Proteomics.

[B6] Kalita B, Patra A, Mukherjee AK (2017). Unraveling the proteome composition and immuno-profiling of
Western India Russell’s viper venom for in-depth understanding of its
pharmacological properties, clinical manifestations, and effective antivenom
treatment. J Proteome Res.

[B7] Mukherjee AK, Kalita B, Mackessy SP (2016). A proteomic analysis of Pakistan Daboia russelii russelii venom
and assessment of potency of Indian polyvalent and monovalent
antivenom. J Proteomics.

[B8] Tan NH, Fung SY, Tan KY, Yap MKK, Gnanathasan CA, Tan CH (2015). Functional venomics of the Sri Lankan Russell's viper (Daboia
russelii) and its toxinological correlations. J Proteomics.

[B9] Belt PJ, Malhotra A, Thorpe RS, Warrell DA, Wuster W (1997). 16 Russell's viper in Indonesia: snakebite and systematics.

[B10] Kularatne SA (2003). Epidemiology and clinical picture of the Russell's viper (Daboia
russelii russelii) bite in Anuradhapura, Sri Lanka: a prospective study of
336 patients. Southeast Asian J Trop Med Public Health.

[B11] Silva A, Maduwage K, Sedgwick M, Pilapitiya S, Weerawansa P, Dahanayaka NJ (2016). Neurotoxicity in Russell's viper (Daboia russelii) envenoming in
Sri Lanka: a clinical and neurophysiological study. Clin Toxicol (Phila).

[B12] Swe TN, Khin M, Thwin MM, Naing S (1997). Acute changes in serum cortisol levels following Russel's viper
bites in Myanmar. Southeast Asian J Trop Med Public Health.

[B13] Tun P, Phillips RE, Warrell DA, Moore RA, Tin Nu S, Myint L (1987). Acute and chronic pituitary failure resembling Sheehan's syndrome
following bites by Russell's viper in Burma. Lancet.

[B14] Hung DZ, Wu ML, Deng JF, Lin-Shiau SY (2002). Russell's viper snakebite in Taiwan: differences from other Asian
countries. Toxicon.

[B15] Li Q, Gong J, Wei Y, Zhao X (2004). Report of bite of Vipera russelii siamensis causes severe
pulmonary hemorrhage. J Snake.

[B16] Lu X, Gong FY, Xu YS, Yan J, Xiao SW (2015). A case report of cerebral infarction after cerebral hemorrhage
caused by Vipera russelii siamensis bite. J Snake.

[B17] Sanz L, Quesada-Bernat S, Chen PY, Lee CD, Chiang JR, Calvete JJ (2018). Translational venomics: third-generation antivenomics of
anti-siamese Russell's viper, Daboia siamensis, antivenom manufactured in
Taiwan CDC's Vaccine Center. Trop Med Infect Dis.

[B18] Tan KY, Tan NH, Tan CH (2018). Venom proteomics and antivenom neutralization for the Chinese
eastern Russell's viper, Daboia siamensis from Guangxi and
Taiwan. Sci Rep.

[B19] Risch M, Georgieva D, von Bergen M, JehmLich N, Genov N, Arni RK (2009). Snake venomics of the Siamese Russell's viper (Daboia russelli
siamensis) -- relation to pharmacological activities. J Proteomics.

[B20] Adiwinata R, Nelwan EJ (2015). Snakebite in Indonesia. Acta Med Indones.

[B21] Tan CH, Liew JL, Tan KY, Tan NH (2016). Assessing SABU (Serum Anti Bisa Ular), the sole Indonesian
antivenom: A proteomic analysis and neutralization efficacy
study. Sci Rep.

[B22] Lingam TMC, Tan KY, Tan CH (2019). Thai Russell's viper monospecific antivenom is immunoreactive and
effective in neutralizing the venom of Daboia siamensis from Java,
Indonesia. Toxicon.

[B23] Tan CH, Tan KY, Tan NH (2019). A protein decomplexation strategy in snake venom
proteomics. Methods Mol Biol.

[B24] LaemmLi UK (1970). Cleavage of structural proteins during the Assembly of the head
of bacteriophage T4. Nature.

[B25] Tan CH, Tan KY, Tan NH (2016). Revisiting Notechis scutatus venom: on shotgun proteomics and
neutralization by the “bivalent” Sea Snake Antivenom. J Proteomics.

[B26] Chong HP, Tan KY, Tan NH, Tan CH (2019). Exploring the diversity and novelty of toxin genes in Naja
sumatrana, the equatorial spitting cobra from Malaysia through De Novo
venom-gland transcriptomics. Toxins (Basel).

[B27] Tan KY, Tan CH, Chanhome L, Tan NH (2017). Comparative venom gland transcriptomics of Naja kaouthia
(monocled cobra) from Malaysia and Thailand: elucidating geographical venom
variation and insights into sequence novelty. PeerJ.

[B28] Tan CH, Tan KY, Yap MKK, Tan NH (2017). Venomics of Tropidolaemus wagleri, the sexually dimorphic temple
pit viper: unveiling a deeply conserved atypical toxin
arsenal. Sci Rep.

[B29] Tan CH, Tan KY, Ng TS, Quah ESH, Ismail AK, Khomvilai S (2019). Venomics of Trimeresurus (Popeia) nebularis, the Cameron
Highlands pit viper from Malaysia: Insights into venom proteome, toxicity
and neutralization of antivenom. Toxins (Basel).

[B30] Wong KY, Tan CH, Tan KY, Quraishi NH, Tan NH (2018). Elucidating the biogeographical variation of the venom of Naja
naja (spectacled cobra) from Pakistan through a venom-decomplexing proteomic
study. J Proteomics.

[B31] Lambeau G, Ancian P, Nicolas JP, Cupillard L, Zvaritch E, Lazdunski M (1996). A family of receptors for secretory phospholipases
A2. C R Seances Soc Biol Fil.

[B32] Tsai IH, Tsai HY, Wang YM, Tun P, Warrell DA (2007). Venom phospholipases of Russell's vipers from Myanmar and eastern
India--cloning, characterization and phylogeographic
analysis. Biochim Biophys Acta.

[B33] Kini RM (2003). Excitement ahead: structure, function and mechanism of snake
venom phospholipase A2 enzymes. Toxicon.

[B34] Sharma M, Das D, Iyer JK, Kini RM, Doley R (2015). Unveiling the complexities of Daboia russelii venom, a medically
important snake of India, by tandem mass spectrometry. Toxicon.

[B35] Wang YM, Lu PJ, Ho CL, Tsai IH (1992). Characterization and molecular cloning of neurotoxic
phospholipases A2 from Taiwan viper (Vipera russelli
formosensis). Eur J Biochem.

[B36] WHO (2016). Guidelines for the management of snake-bites.

[B37] Alirol E, Sharma SK, Bawaskar HS, Kuch U, Chappuis F (2010). Snake bite in South Asia: a review. PLoS Negl Trop Dis.

[B38] Phillips RE, Theakston RD, Warrell DA, Galigedara Y, Abeysekera DT, Dissanayaka P (1988). Paralysis, rhabdomyolysis and haemolysis caused by bites of
Russell's viper (Vipera russelli pulchella) in Sri Lanka: failure of Indian
(Haffkine) antivenom. Q J Med.

[B39] You SQ, Chen LP (2007). Treatment on patients with Vipera russelii siamensis
bites. J Clin Exp Med.

[B40] Mukherjee AK, Mackessy SP (2014). Pharmacological properties and pathophysiological significance of
a Kunitz-type protease inhibitor (Rusvikunin-II) and its protein complex
(Rusvikunin complex) purified from Daboia russelii russelii
venom. Toxicon.

[B41] Slagboom J, Kool J, Harrison RA, Casewell NR (2017). Haemotoxic snake venoms: their functional activity, impact on
snakebite victims and pharmaceutical promise. Br J Haematol.

[B42] Kini RM (2005). Serine proteases affecting blood coagulation and fibrinolysis
from snake venoms. Pathophysiol Haemost Thromb.

[B43] Braud S, Bon C, Wisner A (2000). Snake venom proteins acting on hemostasis. Biochimie.

[B44] Nakayama D, Ben Ammar Y, Miyata T, Takeda S (2011). Structural basis of coagulation factor V recognition for cleavage
by RVV-V. FEBS Lett.

[B45] Tans G, Rosing J (2001). Snake venom activators of factor X: an overview. Haemostasis.

[B46] Ogawa T, Chijiwa T, Oda-Ueda N, Ohno M (2005). Molecular diversity and accelerated evolution of C-type
lectin-like proteins from snake venom. Toxicon.

[B47] Calvete JJ, Marcinkiewicz C, Monleon D, Esteve V, Celda B, Juarez P (2005). Snake venom disintegrins: evolution of structure and
function. Toxicon.

[B48] Sajevic T, Leonardi A, Križaj I (2011). Haemostatically active proteins in snake venoms. Toxicon.

[B49] Tan NH, Tan CH, Utkin Y.N., Krivoshein A.V. (2016). Cytotoxicity of snake venoms and toxins: mechanisms and
applications. Snake Venoms and Envenomation. Modern Trends and Future
Prospects.

[B50] Lavin MF, Earl S, Birrell G, St. Pierre L, Guddat LW, Jd Jersey, Mackessy SP (2010). Snake venom nerve growth factors. Handbook of Venoms and Toxins of Reptiles.

[B51] Dhananjaya BL, D´souza CJM (2010). An overview on nucleases (DNase, RNase, and phosphodiesterase) in
snake venoms. Biochemistry (Mosc).

[B52] Williams DJ, Faiz MA, Abela-Ridder B, Ainsworth S, Bulfone TC, Nickerson AD (2019). Strategy for a globally coordinated response to a priority
neglected tropical disease: snakebite envenoming. PLoS Negl Trop Dis.

[B53] Oh AMF, Tan CH, Tan KY, Quraishi NH, Tan NH (2019). Venom proteome of Bungarus sindanus (Sind krait) from Pakistan
and in vivo cross-neutralization of toxicity using an Indian polyvalent
antivenom. J Proteomics.

